# Supplementary material for: Novel compounds derived from AR-12 that demonstrate host-directed clearance of intracellular Salmonella enterica Serovar Typhimurium
Source: bioRxiv. 2026 May 22:2025.12.30.696991. Preprint. [Version 2] doi: 10.64898/2025.12.30.696991 (PMC13228410; doi:10.64898/2025.12.30.696991)
Supplement: Supplement 1 — Supplemental Table 1. Chemical structures of AR-12 analogs screened for host-directed therapy against intracellular Salmonella infection. Parent compound AR-12 provided for reference. Supplemental Table 2. Compounds with direct activity against planktonic Salmonella. Concentration at which intracellular susceptible S. Typhimurium burden is 50% in RAW 264.7 macrophages determined by gentamicin protection assay (Susc. IC50) and concentration where RAW 264.7 macrophage cell viability is reduced by 50% (LC50) as determined by MTT assay. The concentration of compounds that reduce planktonic susceptible S. Typhimurium viability to 50% as measured by optical density (MIC50). Selectivity of each compound calculated by LC50 / IC50. Host-directed therapeutic (HDT) ratio calculated by MIC50 / IC50. Supplemental Figure 1. Venn diagram demonstrating n=81 compound potency against intracellular susceptible S. Typhimurium and cytotoxicity against RAW 264.7 host cell relative to parental compound AR-12. Supplemental Figure 2: Evaluating lead compound efficacy against MDR S. Typhimurium. A) Schematic illustrating gentamicin protection assay. Day 0 RAW 264.7 macrophages are seeded into 96-well plate and STM culture is inoculated. Day 1: RAW 264.7 cells are infected with STM at an MOI of 10 for 30 min, washed, then treated with gentamicin for 1hr to remove extracellular bacteria. AR-12 analogs added to infected cells for 22hr incubation. Day 2: RAWs are washed, lysed, diluted and then dropped onto agar plates. Day 3: colony forming units (CFUs) counted. B) Dose effect curves of intracellular MDR S. Typhimurium viability from RAW 264.7 macrophages after 22hr treatment with hit compounds. CFUs normalized to untreated controls. Parental compound AR-12 included for comparison. Supplemental Figure 3. A) Chemical structure of 424 which was conjugated to functionalized agarose bead for affinity capture proteomic analysis for 370. B) Chemical structure of 490 which was conjugated to functional [file media-1.docx]

**Supplemental Information:**

**Novel compounds derived from AR-12 that demonstrate host-directed clearance of intracellular *Salmonella enterica* Serovar Typhimurium**

Elizabeth G. Graham-Gurysh^a†^, M. Shamim Hasan Zahid^a†^, Devika M. Varma^a^, Antonio Landavazo^b^, Ojas A Namjoshi ^b^, Joseph W Wilson ^b^, Monica M. Johnson^a^, Ryan N. Woodring^a^, Aaron T. Hendricksen^a^, Joseph Vath^a^, Erica N. Pino^a^, Eric M. Bachelder^a^, Bruce E. Blough^b^, Kristy M. Ainslie^acd^*

^a^ Division of Pharmacoengineering and Molecular Pharmaceutics, Eshelman School of Pharmacy, University of North Carolina at Chapel Hill, Chapel Hill, NC, USA

^b^ Center for Drug Discovery, RTI International, Research Triangle Park, Durham, NC, USA

^c^ Joint Department of Biomedical Engineering, University of North Carolina at Chapel Hill and North Carolina State University, Chapel Hill, NC, USA

^d^ Department of Microbiology and Immunology, UNC School of Medicine, University of North Carolina, Chapel Hill, NC, USA

**^†^**These authors contributed equally to this work

*Corresponding Author

Kristy M. Ainslie

Professor

Division of Pharmacoengineering and Molecular Pharmaceutics

UNC Eshelman School of Pharmacy

4012 Marsico Hall, 125 Mason Farm Road

Chapel Hill, NC 27599, United States

ainsliek@email.unc.edu

**Supplemental Table 1.** Chemical structures of AR-12 analogs screened for host-directed therapy against intracellular *Salmonella* infection. Parent compound AR-12 provided for reference.

| **AR--12** | **202** | **203** | **229** | **230** | **232** | **247** | **285** |
| --- | --- | --- | --- | --- | --- | --- | --- |
|  |  |  |  |  |  |  |  |
| **286** | **312** | **313** | **314** | **315** | **316** | **317** | **318** |
|  |  |  |  |  |  |  |  |
| **319** | **321** | **322** | **323** | **324** | **327** | **330** | **334** |
|  |  |  |  |  |  |  |  |
| **336** | **337** | **338** | **339** | **340** | **341** | **352** | **353** |
|  |  |  |  |  |  |  |  |
| **354** | **355** | **356** | **357** | **358** | **362** | **363** | **364** |
|  |  |  |  |  |  |  |  |

| **365** | **366** | **367** | **368** | **370** | **371** | **372** | **373** |
| --- | --- | --- | --- | --- | --- | --- | --- |
|  |  |  |  |  |  |  |  |
| **374** | **375** | **376** | **377** | **378** | **381** | **389** | **392** |
|  |  |  |  |  |  |  |  |
| **394** | **395** | **396** | **397** | **412** | **413** | **414** | **415** |
|  |  |  |  |  |  |  |  |
| **416** | **417** | **418** | **419** | **420** | **421** | **422** | **423** |
|  |  |  |  |  |  |  |  |
| **424** | **425** | **426** | **427** | **428** | **429** | **430** | **431** |
|  |  |  |  |  |  |  |  |

| **432** | **433** |
| --- | --- |
|  |  |

**Supplemental Table 2.** **Compounds with direct activity against planktonic *Salmonella*.** Concentration at which intracellular susceptible *S.* Typhimurium burden is 50% in RAW 264.7 macrophages determined by gentamicin protection assay (Susc. IC_50_) and concentration where RAW 264.7 macrophage cell viability is reduced by 50% (LC_50_) as determined by MTT assay. The concentration of compounds that reduce planktonic susceptible *S*. Typhimurium viability to 50% as measured by optical density (MIC_50_). Selectivity of each compound calculated by LC_50_ / IC_50_. Host-directed therapeutic (HDT) ratio calculated by MIC_50_ / IC_50_.

| **Compound** | **Susc.**  **IC_50_ (µM)** | **Host Cell**  **LC_50_ (µM)** | **Susc.**  **MIC_50_ (µM)** | **Selectivity (LC_50_ / IC_50_)** | **HDT Ratio**  **(MIC_50_ / IC_50_)** |  |
| --- | --- | --- | --- | --- | --- | --- |
| 416 | 0.21 | 8.1 | 16.6 | 38 | 79 |  |
| 418 | 0.12 | 26.4 | 16.4 | 225 | 140 |  |
| 420 | 0.36 | 6.2 | 17.0 | 17 | 47 |  |
| 424 | 0.17 | 1.8 | 19.7 | 10 | 116 |  |
| 425 | 0.23 | 1.9 | 10.5 | 8 | 46 |  |
| 429 | 1.33 | 2.2 | 14.4 | 2 | 11 |  |
| 433 | 0.39 | 8.2 | 17.2 | 21 | 44 |  |


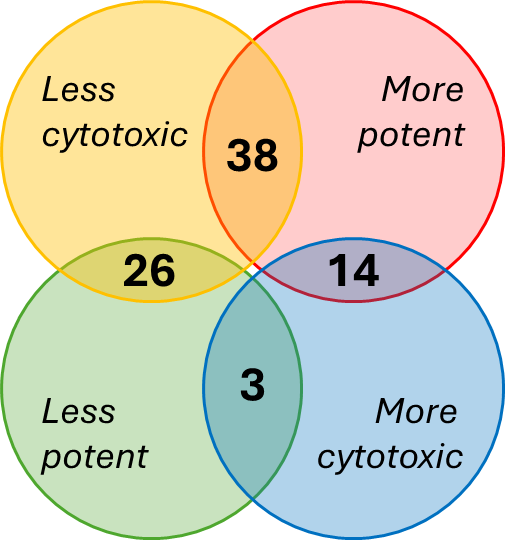


**Supplemental Figure 1.** Venn diagram demonstrating n=81 compound potency against intracellular susceptible *S.* Typhimurium and cytotoxicity against RAW 264.7 host cell relative to parental compound AR-12.


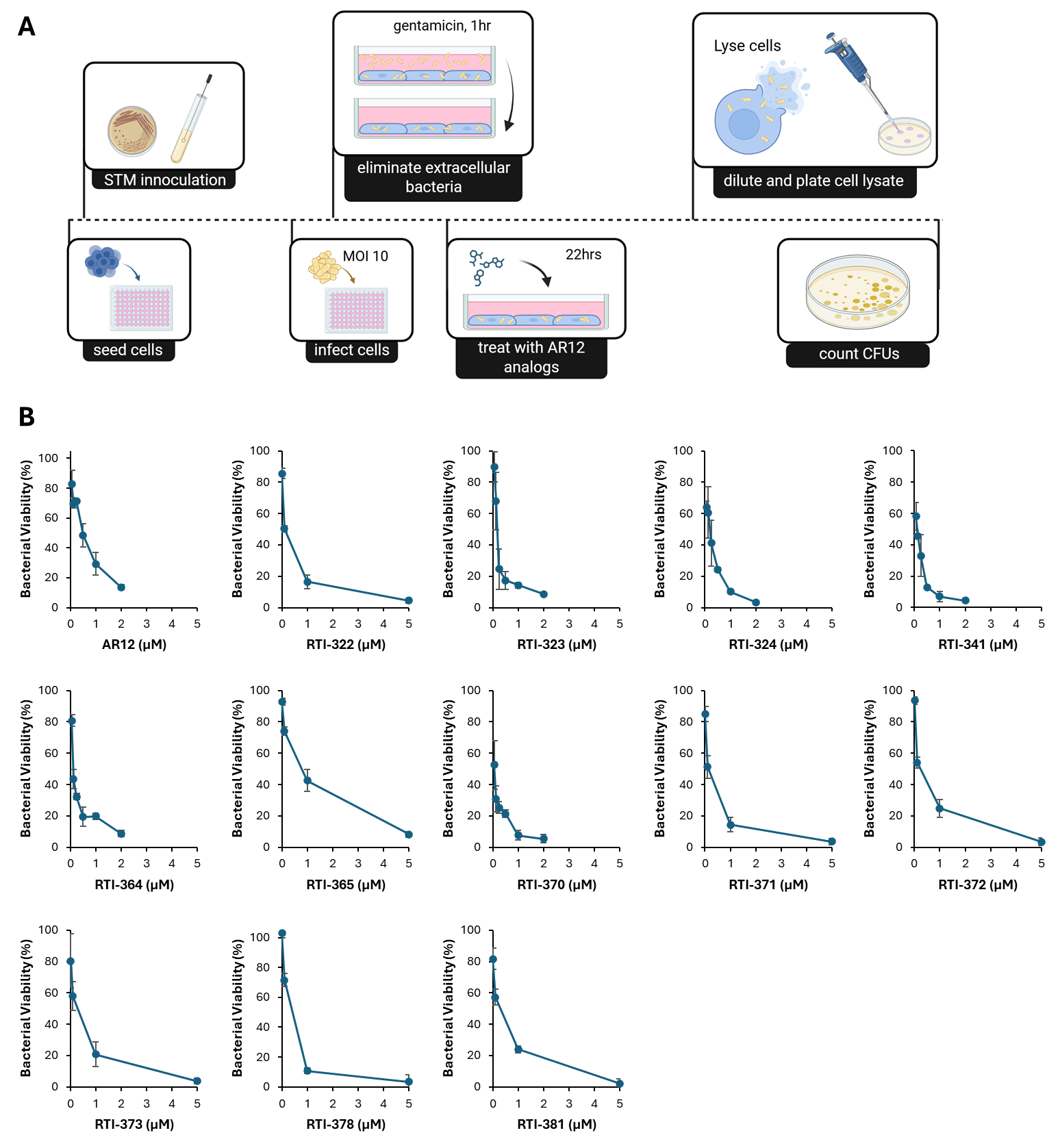


**Supplemental Figure 2: Evaluating lead compound efficacy against MDR *S*. Typhimurium**. **A)** Schematic illustrating gentamicin protection assay. Day 0 RAW 264.7 macrophages are seeded into 96-well plate and STM culture is inoculated. Day 1: RAW 264.7 cells are infected with STM at an MOI of 10 for 30 min, washed, then treated with gentamicin for 1hr to remove extracellular bacteria. AR-12 analogs added to infected cells for 22hr incubation. Day 2: RAWs are washed, lysed, diluted and then dropped onto agar plates. Day 3: colony forming units (CFUs) counted. **B)** Dose effect curves of intracellular MDR *S.* Typhimurium viability from RAW 264.7 macrophages after 22hr treatment with hit compounds. CFUs normalized to untreated controls. Parental compound AR-12 included for comparison.


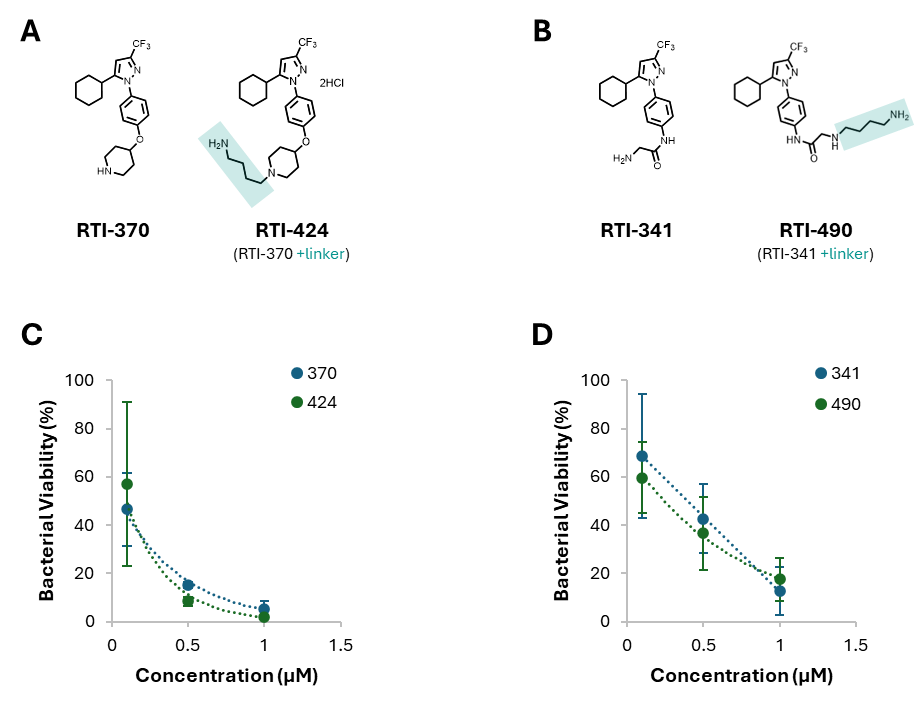


**Supplemental Figure 3.** **A)** Chemical structure of 424 which was conjugated to functionalized agarose bead for affinity capture proteomic analysis for 370. **B)** Chemical structure of 490 which was conjugated to functionalized agarose bead for affinity capture proteomic analysis for 341. **C-D)** Dose effect curves of intracellular susceptible *S.* Typhimurium viability from RAW 264.7 macrophages after 22hr treatment with lead compounds and their ligated counterparts. CFUs normalized to untreated controls.


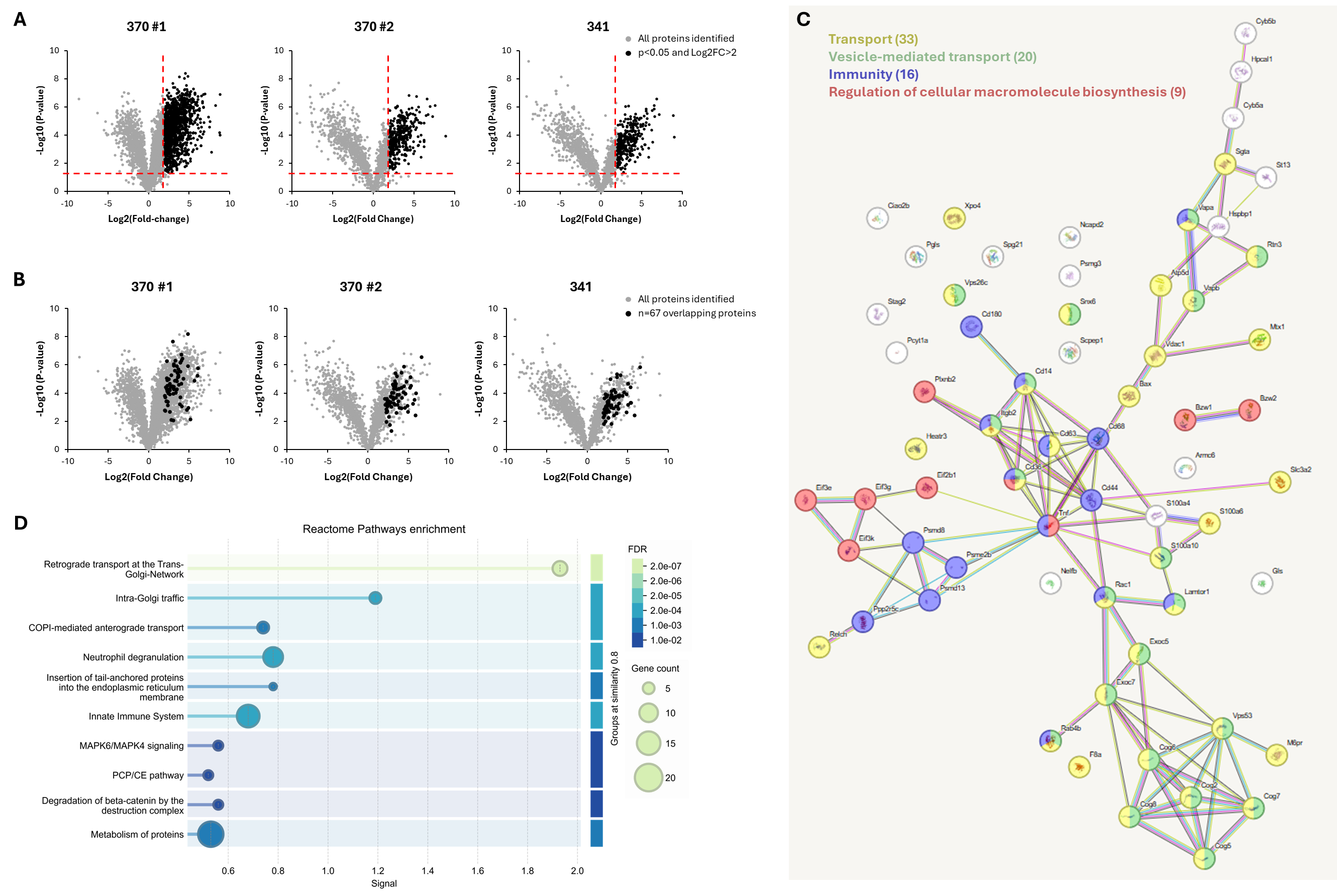


**Supplemental Figure 4.** **A)** Volcano plots illustrating significant proteins identified by affinity capture experiments. Compound 370 was evaluated twice (two separate experiments: 370 #1 and 370 #2) and 341 was evaluated once. **B)** Volcano plots illustrating overlapping proteins identified across all three affinity capture experiments. **C)** STRING (Search Tool for the Retrieval of Interacting Genes/Proteins) network of n=67 overlapping proteins identified by affinity capture across all three experiments. **D)** Reactome pathway enrichment identified by STRING database.

**Supplemental Table 3.** Overlapping proteins (n=80) identified in all three affinity capture proteomic screens. Log2 fold change (Log2FC) compared to bead control and -Log10(p-value) for each experiment listed.

|  |  | **370 #1** | | **370 #2** | | **341** | |
| --- | --- | --- | --- | --- | --- | --- | --- |
| **Accession** | **Description** | **Log2 FC** | **-Log10 (p-value)** | **Log2 FC** | **-Log10 (p-value)** | **Log2 FC** | **-Log10 (p-value)** |
| Q9CQV8 | 14-3-3 protein beta/alpha OS=Mus musculus OX=10090 GN=Ywhab PE=1 SV=3 | 6.66 | 6.29 | 2.41 | 3.15 | 2.25 | 2.12 |
| P62259 | 14-3-3 protein epsilon OS=Mus musculus OX=10090 GN=Ywhae PE=1 SV=1 | 5.91 | 6.26 | 4.00 | 3.51 | 2.69 | 3.06 |
| Q9WVJ2 | 26S proteasome non-ATPase regulatory subunit 13 OS=Mus musculus OX=10090 GN=Psmd13 PE=1 SV=1 | 4.87 | 8.18 | 2.81 | 3.33 | 2.43 | 2.91 |
| Q9CX56 | 26S proteasome non-ATPase regulatory subunit 8 OS=Mus musculus OX=10090 GN=Psmd8 PE=1 SV=2 | 3.66 | 5.71 | 3.22 | 4.30 | 2.27 | 2.85 |
| Q00558 | 40-kDa huntingtin-associated protein OS=Mus musculus OX=10090 GN=F8a1 PE=1 SV=1 | 3.10 | 4.27 | 2.72 | 1.74 | 2.98 | 1.80 |
| P10852 | 4F2 cell-surface antigen heavy chain OS=Mus musculus OX=10090 GN=Slc3a2 PE=1 SV=1 | 2.91 | 4.47 | 4.43 | 5.85 | 4.11 | 5.27 |
| Q9CQ60 | 6-phosphogluconolactonase OS=Mus musculus OX=10090 GN=Pgls PE=1 SV=1 | 3.71 | 4.60 | 3.06 | 4.62 | 2.86 | 3.64 |
| Q07813 | Apoptosis regulator BAX OS=Mus musculus OX=10090 GN=Bax PE=1 SV=1 | 3.47 | 5.92 | 3.43 | 5.39 | 3.22 | 3.71 |
| Q8BNU0 | Armadillo repeat-containing protein 6 OS=Mus musculus OX=10090 GN=Armc6 PE=1 SV=1 | 3.36 | 4.00 | 3.17 | 3.54 | 2.95 | 2.98 |
| Q91YH5 | Atlastin-3 OS=Mus musculus OX=10090 GN=Atl3 PE=1 SV=1 | 5.35 | 5.39 | 3.83 | 3.44 | 3.79 | 3.22 |
| Q9D3D9 | ATP synthase subunit delta, mitochondrial OS=Mus musculus OX=10090 GN=Atp5f1d PE=1 SV=1 | 2.39 | 3.37 | 4.11 | 4.38 | 3.65 | 5.14 |
| Q9CQC6 | Basic leucine zipper and W2 domain-containing protein 1 OS=Mus musculus OX=10090 GN=Bzw1 PE=1 SV=1 | 3.69 | 5.17 | 3.14 | 4.01 | 2.63 | 3.44 |
| Q91VK1 | Basic leucine zipper and W2 domain-containing protein 2 OS=Mus musculus OX=10090 GN=Bzw2 PE=1 SV=1 | 3.09 | 6.05 | 2.95 | 4.02 | 2.25 | 3.92 |
| P24668 | Cation-dependent mannose-6-phosphate receptor OS=Mus musculus OX=10090 GN=M6pr PE=1 SV=1 | 6.08 | 5.76 | 3.54 | 2.60 | 2.63 | 2.23 |
| Q62192 | CD180 antigen OS=Mus musculus OX=10090 GN=Cd180 PE=1 SV=2 | 3.24 | 4.87 | 5.90 | 2.41 | 5.85 | 2.41 |
| P15379 | CD44 antigen OS=Mus musculus OX=10090 GN=Cd44 PE=1 SV=3 | 2.78 | 2.10 | 4.76 | 4.81 | 4.38 | 3.82 |
| P41731 | CD63 antigen OS=Mus musculus OX=10090 GN=Cd63 PE=1 SV=2 | 2.73 | 4.46 | 5.91 | 4.00 | 4.83 | 3.92 |
| P49586 | Choline-phosphate cytidylyltransferase A OS=Mus musculus OX=10090 GN=Pcyt1a PE=1 SV=1 | 3.03 | 4.35 | 2.13 | 3.15 | 2.49 | 3.38 |
| O35638 | Cohesin subunit SA-2 OS=Mus musculus OX=10090 GN=Stag2 PE=1 SV=3 | 3.95 | 3.71 | 2.28 | 1.96 | 2.25 | 1.97 |
| Q8K2Z4 | Condensin complex subunit 1 OS=Mus musculus OX=10090 GN=Ncapd2 PE=1 SV=2 | 3.61 | 5.14 | 2.25 | 2.84 | 2.43 | 2.72 |
| Q921L5 | Conserved oligomeric Golgi complex subunit 2 OS=Mus musculus OX=10090 GN=Cog2 PE=1 SV=2 | 3.70 | 5.27 | 3.61 | 3.89 | 3.41 | 3.66 |
| Q8C0L8 | Conserved oligomeric Golgi complex subunit 5 OS=Mus musculus OX=10090 GN=Cog5 PE=1 SV=3 | 5.69 | 4.87 | 4.44 | 4.50 | 4.51 | 4.05 |
| Q8R3I3 | Conserved oligomeric Golgi complex subunit 6 OS=Mus musculus OX=10090 GN=Cog6 PE=1 SV=2 | 2.00 | 3.79 | 3.64 | 3.16 | 3.75 | 3.04 |
| Q3UM29 | Conserved oligomeric Golgi complex subunit 7 OS=Mus musculus OX=10090 GN=Cog7 PE=1 SV=1 | 3.45 | 4.92 | 4.96 | 2.67 | 4.82 | 2.61 |
| Q9JJA2 | Conserved oligomeric Golgi complex subunit 8 OS=Mus musculus OX=10090 GN=Cog8 PE=1 SV=3 | 3.08 | 2.06 | 3.87 | 2.95 | 3.59 | 2.56 |
| P56395 | Cytochrome b5 OS=Mus musculus OX=10090 GN=Cyb5a PE=1 SV=2 | 2.18 | 3.42 | 4.37 | 4.06 | 4.01 | 3.85 |
| Q9CQX2 | Cytochrome b5 type B OS=Mus musculus OX=10090 GN=Cyb5b PE=1 SV=1 | 5.15 | 2.13 | 3.60 | 5.20 | 3.01 | 4.12 |
| Q9D187 | Cytosolic iron-sulfur assembly component 2B OS=Mus musculus OX=10090 GN=Ciao2b PE=1 SV=1 | 2.47 | 4.99 | 2.88 | 1.32 | 3.54 | 3.07 |
| Q61753 | D-3-phosphoglycerate dehydrogenase OS=Mus musculus OX=10090 GN=Phgdh PE=1 SV=3 | 2.14 | 3.71 | 2.69 | 3.59 | 2.21 | 3.08 |
| Q8JZQ9 | Eukaryotic translation initiation factor 3 subunit B OS=Mus musculus OX=10090 GN=Eif3b PE=1 SV=1 | 4.52 | 6.28 | 3.00 | 6.06 | 3.04 | 6.02 |
| P60229 | Eukaryotic translation initiation factor 3 subunit E OS=Mus musculus OX=10090 GN=Eif3e PE=1 SV=1 | 4.32 | 5.03 | 3.56 | 4.27 | 3.50 | 3.74 |
| Q9Z1D1 | Eukaryotic translation initiation factor 3 subunit G OS=Mus musculus OX=10090 GN=Eif3g PE=1 SV=2 | 3.52 | 4.85 | 3.30 | 4.85 | 3.28 | 4.24 |
| Q9QZD9 | Eukaryotic translation initiation factor 3 subunit I OS=Mus musculus OX=10090 GN=Eif3i PE=1 SV=1 | 4.36 | 5.79 | 3.12 | 5.36 | 3.33 | 5.13 |
| Q9DBZ5 | Eukaryotic translation initiation factor 3 subunit K OS=Mus musculus OX=10090 GN=Eif3k PE=1 SV=1 | 4.03 | 6.72 | 3.37 | 4.47 | 2.35 | 3.75 |
| Q3TPX4 | Exocyst complex component 5 OS=Mus musculus OX=10090 GN=Exoc5 PE=1 SV=2 | 6.01 | 5.23 | 2.50 | 3.30 | 2.32 | 2.77 |
| O35250 | Exocyst complex component 7 OS=Mus musculus OX=10090 GN=Exoc7 PE=1 SV=2 | 4.87 | 5.91 | 2.28 | 3.24 | 2.10 | 3.63 |
| Q9ESJ0 | Exportin-4 OS=Mus musculus OX=10090 GN=Xpo4 PE=1 SV=2 | 2.08 | 2.74 | 5.31 | 4.51 | 5.33 | 4.42 |
| D3Z7P3 | Glutaminase kidney isoform, mitochondrial OS=Mus musculus OX=10090 GN=Gls PE=1 SV=1 | 2.24 | 6.26 | 2.83 | 3.78 | 2.65 | 2.66 |
| P01900 | H-2 class I histocompatibility antigen, D-D alpha chain OS=Mus musculus OX=10090 GN=H2-D1 PE=1 SV=1 | 2.23 | 3.40 | 2.81 | 3.73 | 2.49 | 3.20 |
| P01902 | H-2 class I histocompatibility antigen, K-D alpha chain OS=Mus musculus OX=10090 GN=H2-K1 PE=1 SV=1 | 2.01 | 2.77 | 2.32 | 3.18 | 2.14 | 2.84 |
| Q8BQM4 | HEAT repeat-containing protein 3 OS=Mus musculus OX=10090 GN=Heatr3 PE=1 SV=1 | 2.61 | 2.65 | 3.32 | 3.17 | 3.24 | 2.92 |
| Q9CQN1 | Heat shock protein 75 kDa, mitochondrial OS=Mus musculus OX=10090 GN=Trap1 PE=1 SV=1 | 2.29 | 5.19 | 2.57 | 3.82 | 2.47 | 3.49 |
| P62748 | Hippocalcin-like protein 1 OS=Mus musculus OX=10090 GN=Hpcal1 PE=1 SV=2 | 3.94 | 6.17 | 5.22 | 4.38 | 3.91 | 3.73 |
| Q99L47 | Hsc70-interacting protein OS=Mus musculus OX=10090 GN=St13 PE=1 SV=1 | 2.81 | 5.28 | 5.25 | 5.11 | 5.03 | 4.64 |
| Q99P31 | Hsp70-binding protein 1 OS=Mus musculus OX=10090 GN=Hspbp1 PE=1 SV=1 | 3.55 | 5.45 | 2.46 | 3.08 | 2.00 | 2.60 |
| Q9EPL8 | Importin-7 OS=Mus musculus OX=10090 GN=Ipo7 PE=1 SV=2 | 2.16 | 5.92 | 3.80 | 4.66 | 3.82 | 4.23 |
| P11835 | Integrin beta-2 OS=Mus musculus OX=10090 GN=Itgb2 PE=1 SV=2 | 2.65 | 4.19 | 2.27 | 2.63 | 2.02 | 2.26 |
| Q6PB66 | Leucine-rich PPR motif-containing protein, mitochondrial OS=Mus musculus OX=10090 GN=Lrpprc PE=1 SV=2 | 2.28 | 4.56 | 2.32 | 2.05 | 2.39 | 2.34 |
| P31996 | Macrosialin OS=Mus musculus OX=10090 GN=Cd68 PE=1 SV=1 | 2.34 | 4.12 | 2.32 | 3.60 | 2.24 | 2.95 |
| Q9CQC8 | Maspardin OS=Mus musculus OX=10090 GN=Spg21 PE=1 SV=1 | 2.22 | 5.06 | 2.57 | 3.42 | 2.54 | 2.77 |
| P47802 | Metaxin-1 OS=Mus musculus OX=10090 GN=Mtx1 PE=1 SV=1 | 4.06 | 4.66 | 3.84 | 4.31 | 3.46 | 4.06 |
| P10810 | Monocyte differentiation antigen CD14 OS=Mus musculus OX=10090 GN=Cd14 PE=1 SV=1 | 3.14 | 5.95 | 5.54 | 3.23 | 5.37 | 3.30 |
| Q8C4Y3 | Negative elongation factor B OS=Mus musculus OX=10090 GN=Nelfb PE=1 SV=2 | 2.37 | 3.12 | 2.38 | 3.05 | 2.12 | 2.43 |
| Q922L6 | Negative elongation factor D OS=Mus musculus OX=10090 GN=Nelfcd PE=1 SV=2 | 2.45 | 3.96 | 5.54 | 3.89 | 5.12 | 3.76 |
| Q08857 | Platelet glycoprotein 4 OS=Mus musculus OX=10090 GN=Cd36 PE=1 SV=2 | 2.42 | 3.97 | 4.91 | 3.11 | 5.02 | 3.18 |
| B2RXS4 | Plexin-B2 OS=Mus musculus OX=10090 GN=Plxnb2 PE=1 SV=1 | 3.29 | 6.29 | 3.18 | 4.48 | 2.63 | 3.28 |
| P17918 | Proliferating cell nuclear antigen OS=Mus musculus OX=10090 GN=Pcna PE=1 SV=2 | 4.70 | 6.30 | 2.32 | 3.11 | 2.20 | 2.70 |
| P97372 | Proteasome activator complex subunit 2 OS=Mus musculus OX=10090 GN=Psme2 PE=1 SV=4 | 2.28 | 4.52 | 3.14 | 3.64 | 2.97 | 3.12 |
| Q9CZH3 | Proteasome assembly chaperone 3 OS=Mus musculus OX=10090 GN=Psmg3 PE=1 SV=1 | 3.49 | 4.28 | 2.89 | 3.01 | 2.28 | 2.81 |
| P08207 | Protein S100-A10 OS=Mus musculus OX=10090 GN=S100a10 PE=1 SV=2 | 2.09 | 3.71 | 4.15 | 5.44 | 2.97 | 3.79 |
| P07091 | Protein S100-A4 OS=Mus musculus OX=10090 GN=S100a4 PE=1 SV=1 | 4.07 | 6.31 | 3.81 | 3.88 | 2.33 | 3.07 |
| P14069 | Protein S100-A6 OS=Mus musculus OX=10090 GN=S100a6 PE=1 SV=3 | 3.24 | 5.60 | 4.61 | 3.93 | 3.26 | 3.54 |
| Q148V7 | RAB11-binding protein RELCH OS=Mus musculus OX=10090 GN=Relch PE=1 SV=1 | 4.75 | 2.79 | 2.58 | 2.31 | 2.05 | 1.81 |
| Q9CQ22 | Ragulator complex protein LAMTOR1 OS=Mus musculus OX=10090 GN=Lamtor1 PE=1 SV=1 | 4.11 | 3.06 | 4.14 | 2.92 | 3.83 | 2.88 |
| P63001 | Ras-related C3 botulinum toxin substrate 1 OS=Mus musculus OX=10090 GN=Rac1 PE=1 SV=1 | 2.30 | 4.91 | 2.11 | 3.60 | 2.85 | 4.97 |
| Q91ZR1 | Ras-related protein Rab-4B OS=Mus musculus OX=10090 GN=Rab4b PE=1 SV=2 | 2.57 | 4.26 | 2.65 | 3.20 | 2.66 | 3.18 |
| Q9ES97 | Reticulon-3 OS=Mus musculus OX=10090 GN=Rtn3 PE=1 SV=2 | 3.90 | 5.52 | 3.11 | 3.69 | 2.56 | 3.13 |
| Q99P72 | Reticulon-4 OS=Mus musculus OX=10090 GN=Rtn4 PE=1 SV=2 | 4.61 | 6.53 | 2.01 | 3.85 | 2.06 | 3.35 |
| Q920A5 | Retinoid-inducible serine carboxypeptidase OS=Mus musculus OX=10090 GN=Scpep1 PE=1 SV=2 | 4.15 | 6.43 | 3.46 | 3.66 | 2.65 | 3.33 |
| Q60996 | Serine/threonine-protein phosphatase 2A 56 kDa regulatory subunit gamma isoform OS=Mus musculus OX=10090 GN=Ppp2r5c PE=1 SV=2 | 4.91 | 2.96 | 5.75 | 3.53 | 5.44 | 3.31 |
| Q76MZ3 | Serine/threonine-protein phosphatase 2A 65 kDa regulatory subunit A alpha isoform OS=Mus musculus OX=10090 GN=Ppp2r1a PE=1 SV=3 | 2.59 | 4.10 | 3.94 | 3.45 | 3.48 | 3.86 |
| Q8BJU0 | Small glutamine-rich tetratricopeptide repeat-containing protein alpha OS=Mus musculus OX=10090 GN=Sgta PE=1 SV=2 | 3.02 | 7.67 | 3.21 | 3.25 | 2.39 | 2.62 |
| Q6P8X1 | Sorting nexin-6 OS=Mus musculus OX=10090 GN=Snx6 PE=1 SV=2 | 2.23 | 4.27 | 4.58 | 2.96 | 3.30 | 2.42 |
| Q99LC8 | Translation initiation factor eIF-2B subunit alpha OS=Mus musculus OX=10090 GN=Eif2b1 PE=1 SV=1 | 2.64 | 4.67 | 3.08 | 4.59 | 2.98 | 3.89 |
| P06804 | Tumor necrosis factor OS=Mus musculus OX=10090 GN=Tnf PE=1 SV=2 | 2.10 | 4.41 | 2.38 | 2.71 | 2.38 | 2.64 |
| O35075 | Vacuolar protein sorting-associated protein 26C OS=Mus musculus OX=10090 GN=Vps26c PE=1 SV=1 | 2.65 | 3.92 | 2.43 | 3.45 | 2.47 | 3.82 |
| Q8CCB4 | Vacuolar protein sorting-associated protein 53 homolog OS=Mus musculus OX=10090 GN=Vps53 PE=1 SV=1 | 3.41 | 4.22 | 3.46 | 4.64 | 3.65 | 5.33 |
| Q9WV55 | Vesicle-associated membrane protein-associated protein A OS=Mus musculus OX=10090 GN=Vapa PE=1 SV=2 | 3.13 | 4.46 | 4.21 | 3.66 | 3.92 | 3.25 |
| Q9QY76 | Vesicle-associated membrane protein-associated protein B OS=Mus musculus OX=10090 GN=Vapb PE=1 SV=3 | 3.04 | 3.95 | 3.75 | 3.41 | 3.77 | 3.79 |
| Q60932 | Voltage-dependent anion-selective channel protein 1 OS=Mus musculus OX=10090 GN=Vdac1 PE=1 SV=3 | 3.87 | 3.22 | 6.65 | 6.57 | 6.53 | 5.84 |

**Supplemental Table 4.** Proteins (n=13) with high average spectral counts (Ave SC) identified by Contaminant Repository for Affinity Purification Mass Spectrometry Data (CRAPome).

| **Gene Symbol** | **Num of Expt. (found/total)** | **Ave SC** | **Max SC** |  | **Accession**  **Number** | **Protein**  **Description** |
| --- | --- | --- | --- | --- | --- | --- |
| LRPPRC | 249 / 716 | 11.3 | 118.0 |  | Q6PB66 | Leucine-rich PPR motif-containing protein, mitochondrial OS=Mus musculus OX=10090 GN=Lrpprc PE=1 SV=2 |
| YWHAE | 447 / 716 | 10.0 | 81.0 |  | P62259 | 14-3-3 protein epsilon OS=Mus musculus OX=10090 GN=Ywhae PE=1 SV=1 |
| TRAP1 | 478 / 716 | 7.2 | 52.0 |  | Q9CQN1 | Heat shock protein 75 kDa, mitochondrial OS=Mus musculus OX=10090 GN=Trap1 PE=1 SV=1 |
| PCNA | 268 / 716 | 6.2 | 70.0 |  | P17918 | Proliferating cell nuclear antigen OS=Mus musculus OX=10090 GN=Pcna PE=1 SV=2 |
| PHGDH | 394 / 716 | 6.2 | 42.0 |  | Q61753 | D-3-phosphoglycerate dehydrogenase OS=Mus musculus OX=10090 GN=Phgdh PE=1 SV=3 |
| EIF3I | 280 / 716 | 5.9 | 37.0 |  | Q9QZD9 | Eukaryotic translation initiation factor 3 subunit I OS=Mus musculus OX=10090 GN=Eif3i PE=1 SV=1 |
| YWHAB | 368 / 716 | 5.8 | 39.0 |  | Q9CQV8 | 14-3-3 protein beta/alpha OS=Mus musculus OX=10090 GN=Ywhab PE=1 SV=3 |
| NELFCD | 51 / 716 | 5.5 | 29.0 |  | Q922L6 | Negative elongation factor D OS=Mus musculus OX=10090 GN=Nelfcd PE=1 SV=2 |
| IPO7 | 191 / 716 | 5.3 | 19.0 |  | Q9EPL8 | Importin-7 OS=Mus musculus OX=10090 GN=Ipo7 PE=1 SV=2 |
| RTN4 | 169 / 716 | 5.1 | 36.0 |  | Q99P72 | Reticulon-4 OS=Mus musculus OX=10090 GN=Rtn4 PE=1 SV=2 |
| PPP2R1A | 316 / 716 | 5.0 | 35.0 |  | Q76MZ3 | Serine/threonine-protein phosphatase 2A 65 kDa regulatory subunit A alpha isoform OS=Mus musculus OX=10090 GN=Ppp2r1a PE=1 SV=3 |
| EIF3B | 282 / 716 | 5.0 | 41.0 |  | Q8JZQ9 | Eukaryotic translation initiation factor 3 subunit B OS=Mus musculus OX=10090 GN=Eif3b PE=1 SV=1 |
| ATL3 | 1 / 716 | 5.0 | 5.0 |  | Q91YH5 | Atlastin-3 OS=Mus musculus OX=10090 GN=Atl3 PE=1 SV=1 |
